# Super-resolution imaging via aperture modulation and intensity extrapolation

**DOI:** 10.1038/s41598-018-33416-9

**Published:** 2018-10-12

**Authors:** Biao Xu, Zhiqiang Wang, Jinping He

**Affiliations:** 1 National Astronomical Observatories/Nanjing Institute of Astronomical Optics & Technology, Chinese Academy of Sciences, Nanjing, 210042 China; 2 Key Laboratory of Astronomical Optics & Technology, Nanjing Institute of Astronomical Optics & Technology, Chinese Academy of Sciences, Nanjing, 210042 China; 3 University of Chinese Academy of Sciences, Beijing, 100049 China

## Abstract

High-resolution telescopic imaging is of great importance in astronomy. Compared to the complexity and huge cost of constructing extremely-large telescopes, super-resolution technique which breaks the diffraction limit of the imaging system can enhance the spatial resolution with compact setup and low cost. In this paper, a novel super-resolution telescopic imaging method based on aperture modulation and intensity extrapolation is demonstrated, with both simulated and experimental studies performed. The simulation results show that the method can enhance the resolving power of a diffraction-limited telescopic imaging system by >5 times in noise-free case, and the improvement still reaches ~1.8 times with a signal-to-noise ratio of only ~10. The preliminary experimental results show a resolution enhancement of ~1.36 times for the limitations of the experimental setup. Better performance is possible with the images for reconstruction denoised and registered more precisely. The method is also useful in wide-field microscopy.

## Introduction

High-resolution telescopic imaging is a necessity in astronomy, especially in the research of binary stars^1^, exoplanets^2^ and gravitational lenses^3^. Space telescopes or ground-based telescopes with perfect adaptive optics (AO) systems can reach diffraction-limited spatial resolution^4^. To increase the spatial resolution and obtain more details of celestial bodies, astronomers are trying to build larger and larger space and ground-based telescopes, such as James Webb Space Telescope (JWST)^5^ and Thirty Meter Telescope (TMT)^6^. However, the cost of this kind of telescopes mounts to astronomical figures^7,8^, and the instruments for the telescopes will also become complex^9^ and expensive. In contrast, Super-resolution (SR) technologies can break the diffraction limit of the imaging system and enhance the spatial resolution of telescopes with compact setup and low cost, which makes SR telescopic imaging attractive and meaningful.

SR technologies are now common in microscopic imaging^10–12^, with several robust SR techniques: photoactivated localization microscopy (PALM)^10^, stochastic optical reconstruction microscopy (STORM)^11^ and stimulated emission depletion microscopy (STED)^12^. Compared with SR microscopy, SR telescopic imaging, especially SR astronomical imaging, are much more difficult, because it is almost impossible to manipulate the illumination light and control the interactions between the illumination light and the object. However, there are still some works carried out on SR telescopic imaging, and they can be simply classified into two categories: (1) image reconstruction; (2) optical SR.

SR technologies based on image reconstruction usually use a single or a sequence of low-resolution images to produce a high-resolution image. This kind of SR techniques attempt to retrieve the lost image details caused by insufficient sampling of imaging sensor, camera movement, ambient light, imperfect position of CCD camera, and so on^13^. Typically, this kind of SR methods can be divided into three groups: frequency domain methods, spatial domain methods and wavelet domain methods. Several algorithms have been reported in the processing and reconstruction high-resolution astronomical images^14–16^.

In the case of optical SR methods, to our best knowledge, only few efforts are made to transcend the diffraction limit of the imaging systems. A phase mask can help to localize the high frequency information from the objects, hence enhance the spatial resolution of the telescope by nearly 2 times^17^. However, the largely reduced signal intensity will hinder the practical applications of the method in astronomical imaging. A novel concept of SR quantum telescope via photon cloning is also proposed^18–20^. Clones of the incoming photon are used to estimate the true position of the incoming photon on the detector with an increased precision, helping improve the angular resolution by larger than 10 times. However, there is no agreement yet on the best setup of such devices^19^.

In this paper, we present a novel SR telescopic imaging method/technology based on aperture modulation and intensity extrapolation, which is called AMIE for short. AMIE is more likely to be a hybrid method, which involves both optical modulation and image reconstruction. The setup of AMIE is compact and available easily with low cost, moreover, no large signal reduction is involved, which makes AMIE possible for real astronomical imaging.

## Principle of AMIE

**Figure 1:**
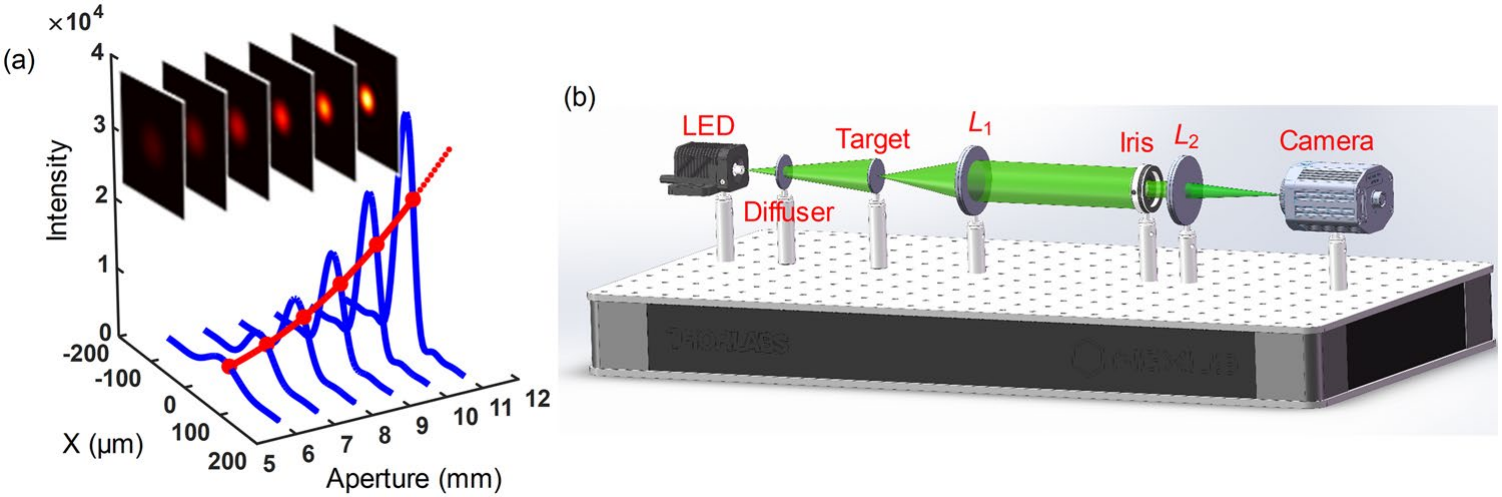
Principle and experimental setup of AMIE. (**a**) Principle of AMIE. The six blue curves show the cross section of six images in the insert, which are the intensity distributions of a point source on the image plane with aperture sizes of 6, 7, 8, 9, 10, 11 mm, respectively. The solid part of the red curve is a fitting of the intensity function on one position of the image plane, and the dashed part is the extrapolation to even larger aperture, which will give the SR information. (**b**) The setup of AMIE imaging system, consisting of a LED source, an optical diffuser, the target, a collimating lens (*L*_1_), a variable iris, an imaging lens (*L*_2_) and a camera.

As shown in Fig. 1(a), the principle of AMIE can be briefly described as follows: (1) A sequence of images are obtained with the same imaging system but different aperture sizes; (2) The image sequence is used to fit the intensity function of the aperture size at each position on the image plane; (3) The fitted intensity function is extrapolated to the aperture size larger than the maximum one of the imaging system; (4) A SR image is reconstructed with the extrapolated intensity values of all position on the image plane. The critical point of the method is how to achieve the fitting process of the intensity function with the image sequence, and the key assumption is that the intensity function is analytic and continuous.

In the case of incoherent imaging of object, the linear, space invariant model for imaging is expressed by^21^.

1 $${{I}}_{{im}}({u},{v})={|{h}({u},{v})|}^{2}\otimes {{I}}_{{g}}({u},{v}),$$

where *u* and *v* are the image plane spatial coordinates, *I*_*g*_ is the ideal geometric irradiance image, ${|h(u,v)|}^{2}$ is commonly known as the *point spread function* (PSF) and ⊗ is the convolution operator. For a telescope with a circular aperture size *D* and focal length *F*, the PSF can be expressed by

2 $${I}({u},{v})={{I}}_{0}{(\frac{2{{J}}_{1}({x})}{{x}})}^{2},$$

where

3 $${x}=\frac{{\pi }D}{{\lambda }}\frac{\sqrt{{{u}}^{2}+{{v}}^{2}}}{\sqrt{{{u}}^{2}+{{v}}^{2}+{{F}}^{2}}}\approx \frac{{\pi }D}{{\lambda }}\frac{\sqrt{{{u}}^{2}+{{v}}^{2}}}{{F}}$$

at paraxial approximation. *J*_1_ is the first order Bessel function of the first kind, *λ* is the wavelength of the source. ${I}_{0}={(\frac{\pi {D}^{2}}{4\lambda F})}^{2}$ is the maximum intensity. Then Eq. (1) can be rewritten as the integral form:

4 $${I}({u},{v})=\frac{{{B}}^{2}}{16\,}\iint {{A}}_{{\xi },{\eta }}{(\frac{2{{J}}_{1}({BD}{{R}}_{{\xi },{\eta }})}{{BD}{{R}}_{{\xi },{\eta }}}{{D}}^{2})}^{2}d{\xi }d{\eta }{,}$$

where

5 $${{A}}_{{\xi },{\eta }}={{I}}_{{g}}({\xi },{\eta }),$$

6 $${B}=\frac{{\pi }}{{\lambda }F\,},$$

7 $${{R}}_{{\xi },{\eta }}=\sqrt{{({u}-{\xi })}^{2}+{({v}-{\eta })}^{2}},$$

with $(\xi ,\eta )$ the geometrical image position of the point sources that compose the object. Commonly, the aperture size of the imaging system is fixed, and Eq. (4) is used for calculating the intensity distribution on the image plane. However, we present a different issue that the aperture size *D* is considered as an independent variable for a fixed position (*p*, *q*) on the image plane. Equation (4) shows that the intensity *I*(*p*, *q*) is a continuous and analytic function of aperture size *D*, and it can be approximated by a discrete summation form as below,

8 $${{I}}_{{p},{q}}({D})\approx {\sum }^{}{{I}}_{{p},{q},{i}}({D}),$$

where

9 $${{I}}_{{p},{q},{i}}({D})=\frac{{{B}}^{2}}{16\,}{{A}}_{{i}}{(\frac{2{{J}}_{1}({BD}{{R}}_{{\boldsymbol{i}}})}{{BD}{{R}}_{{\boldsymbol{i}}}}{{D}}^{2})}^{2}.$$

Here *i* means the *i*-th point source that compose the object. However, the direct use of Eq. (8) as the fitting function seems an impossible task because the object structure is unknown, and the only solution is to assume that each pixel on the image plane is an ideal geometric irradiance image of a point source. To fit the intensity function, i.e. estimate the coefficients $\{{A}_{i}\}$, the number of the images acquired with different aperture sizes should be no less than the total sampling number of the image plane. Moreover, a large number of repeated calculation will be involved when the sampling of the image plane is intensive. A smarter and operable solution is proposed by combining all the point sources that have the same distance *R*_*i*_ away from the position (*p*, *q*) as a term, with the assumption of symmetric PSF. Consequently, both the calculations and the image sequence needed will be reduced greatly. In this paper, we still use Eq. (8) as the fitting function but with a different definition of *i*, which means the *i*-th term instead of the *i*-th point source. The number of the terms is determined by how complex the object is and how accurate the fitting is needed. Then, the extrapolation and SR image reconstruction can be performed with the fitted function *I*_*p*,*q*_(*D*) at each position on the image plane. The details are shown in Methods.

## Experimental setup

Figure 1(b) shows the schematic of the AMIE imaging system, including a LED source with the central wavelength of 530 nm and spectral width of 30 nm (M530F2, Thorlabs), an optical diffuser, the target, a collimating lens (*L*_1_) with focal length of 1 m, a variable iris, an imaging lens (*L*_2_) with focal length of 1 m, and a sCMOS camera (1920 × 1080, pixel size 5.04 µm × 5.04 µm, CS2100M-USB, Thorlabs). The target is set at the front focal plane of *L*_1_ and illuminated by the incoherent LED source, hence it can be treated as infinite far away from the imaging lens *L*_2_. The aperture size of the variable iris can be changed between 1 mm to 12 mm, which is used as the aperture modulator of the imaging system. In order to prevent vignetting and make the setup close to practical situation, the iris is set as close as possible to *L*_2_.

## Results

### Simulation results

**Figure 2:**
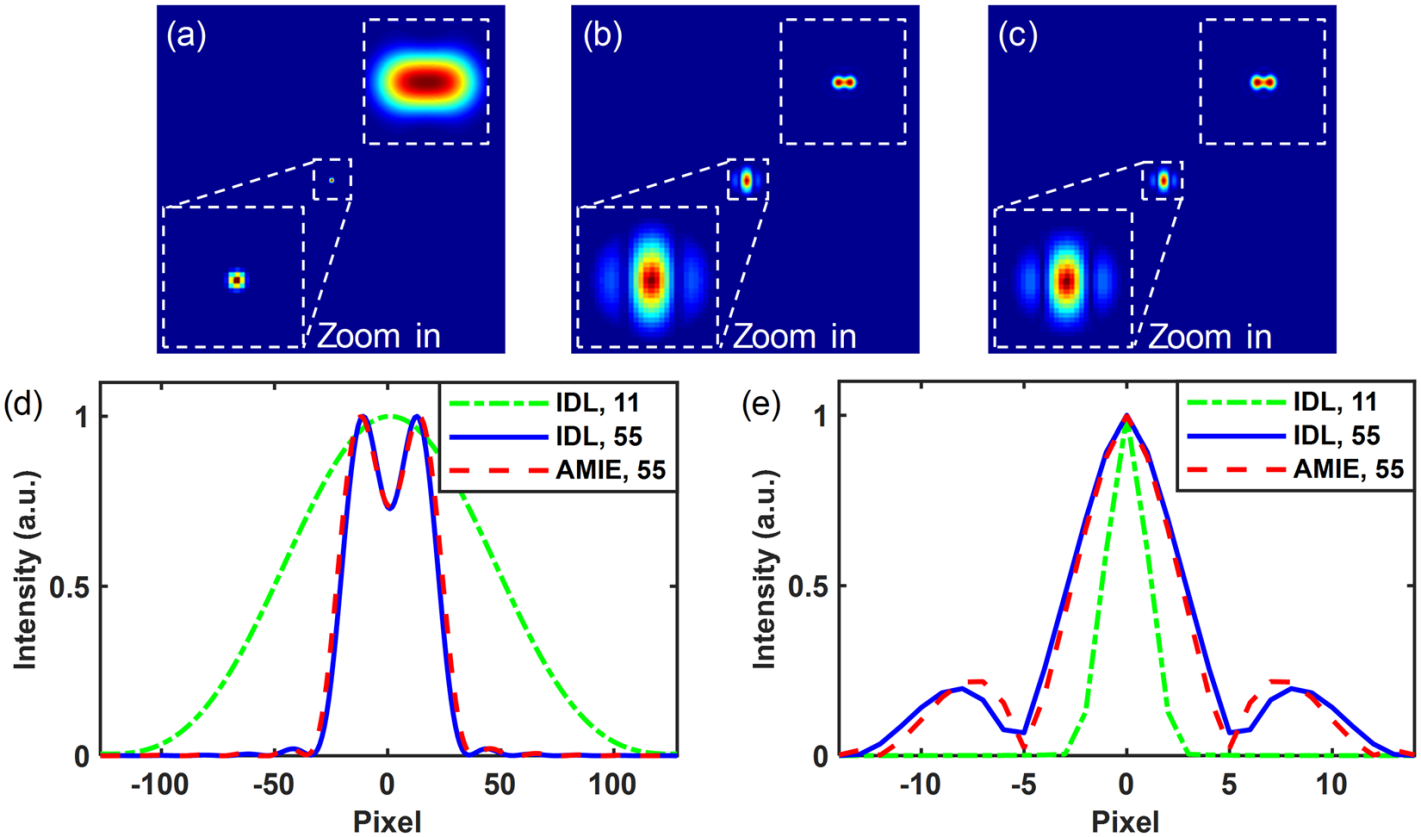
Simulation results of AMIE in the noise-free case. (**a**,**b**) The IDL images with *D* = 11 and 55 mm, respectively. (**c**) The AMIE image with *D*_AMIE_ = 55 mm. (**d**) The cross section comparison of the images in spatial domain in (**a**–**c**). (**e**) The cross section comparison of the Fourier spectra in (**a**–**c**). (The images on the top right of (**a**–**c**) are the corresponding images in spatial domain, and the images in the middle of (**a**–**c**) are in the frequency domain; IDL: the simulated image by ideal diffraction-limited system).

Firstly, we have studied the limit of the resolving power enhancement of AMIE. In this case, the image noise is not considered. A two-point-source object with same brightness is imaged by a diffraction-limited system. The parameters for generating image sequence with Eq. (4) are as follows: ${A}_{\xi ,\eta }$ is 1 at the position of ideal image of the two point sources, and it equals 0 elsewhere, *λ* = 530 nm, and *F* = 1000 mm. The aperture size *D* is changed from 5.5 to 11 mm with a step size of 0.25 mm and 23 images are obtained for SR image reconstruction. We have reduced the center-to-center distance (∆*L*) of the two point sources step by step to check whether AMIE can resolve them without large distortions. All the simulated results can be found in Figs S1–S3 in the Supplementary Information. After careful comparison, we consider the two point sources with ∆*L* = 11.8 µm, which can just be resolved by the imaging system with aperture size *D* = 55 mm according to the Rayleigh criterion (with the saddle-to-peak ratio (SPR) of the intensity distribution of 0.735), may be the critical one. Figure 2(a) shows the images of the two point sources in spatial domain (the one on the top) and frequency domain (the middle one and the zoomed one at the bottom) by ideal diffraction-limited (IDL) system, the two point sources cannot be resolved at all with *D* = 11. With the aperture size increased to 55 mm, the two point sources are just resolved and more high frequency components are obtained, as shown in Fig. 2(b). Figure 2(c) shows the reconstructed image with AMIE, which uses the 23 low-resolution images obtained with aperture size from 5.5 to 11 mm to fit the intensity function, and the function has been extrapolated to aperture size *D*_AMIE_ = 55 mm. To check the difference of the images directly from an IDL system (Fig. 2(b)) and the reconstructed one with AMIE (Fig. 2(c)), we have done the cross section comparison of the images in Fig. 2(a–c) both in spatial domain and in frequency domain, with the results shown in Fig. 2(d–e). The curves demonstrate that the two-point source is evidently unresolved by IDL with *D* = 11 mm and the Fourier spectra is truncated by the cut-off frequency (*f*_cutoff_) in the frequency domain (green dash-dot line). However, with the aperture size extrapolated to *D*_AMIE_ = 55 mm, the same two-point source is successfully resolved and the Fourier spectra clearly transcend the *f*_cutoff_ by the AMIE method (red dashed line). Moreover, the AMIE result agrees well with the IDL ones with *D* = 55 mm (blue solid line) both in the spatial and frequency domain.

Then, we can see that, with the images obtained with aperture sizes no larger than 11 mm, AMIE can resolve the two-point source which can only be resolved by IDL system with *D* = 55 mm and the distortion is negligible, which indicates that AMIE can enhance the resolving power of an IDL system by >5 times in the case of two-point source with equal brightness. Indeed, AMIE also can reconstruct the SR images and resolve the two-point source that can only be resolved by IDL system with *D* > 60 mm, 70 mm, and even 110 mm, as shown in Figs S2,S3 in the Supplementary Information. However, the distortion of the reconstructed SR image becomes a problem with the increased resolving power enhancement. The reason is that the fitting of the intensity function is just an approximation of the true function, and the error will grow when the extrapolation goes too far. In addition, the performance of AMIE of two point sources with non-equal brightness is studied. The results show that AMIE performs well when the difference of the two peaks is not too large, as shown in Fig. S7 in the Supplementary Information.

**Figure 3:**
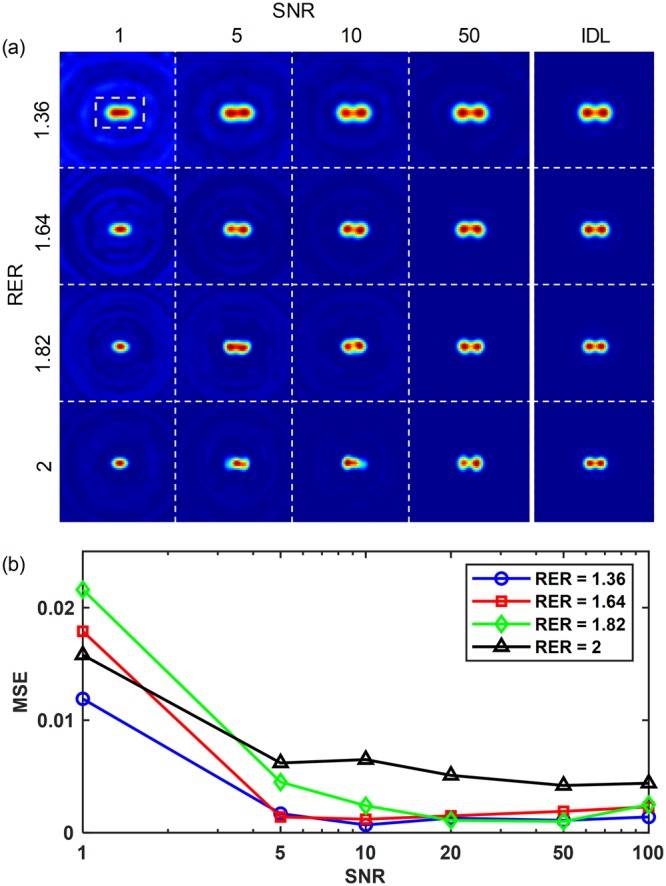
Simulation results of AMIE in the noisy case. (**a**) The AMIE images of two-point sources with different SNRs and RERs. (**b**) The relationship between MSE and SNR with different RERs. (RER: resolution enhancement ratio; SNR: signal-to-noise ratio; MSE: mean squared error).

To investigate the effect of noise on the performance of AMIE, white Gaussian noises are added to the 23 images obtained in the same way as mentioned before. The signal-to-noise ratio (SNR) is defined as the ratio of signal power to the noise power. In practice, the signal power of image decreases with the decrease of aperture size, while the noise power remains the same, thus the SNRs of the sequence of images differ from each other. For the convenient discussion, the SNR mentioned in this paragraph and in Fig. 3 refers in particular to SNR of the image obtained with the maximum aperture *D* = 11 mm. To give a direct view of the influence of noise on the distortion of the reconstructed SR image and the limit of the resolving power enhancement, we use a new parameter RER (resolution enhancement ratio), which is defined by the ratio of spatial resolutions (the spatial resolution can be evaluated by the Rayleigh criterion ∆*d* = 1.22 *λF*/*D*, thus RER = ∆*d*_telescope_/∆*d*_AMIE_ = *D*_AMIE_/*D*_telescope_). We have reconstructed a series of SR images of the two-point sources with different SNRs and different distances. Figure 3(a) shows the AMIE images with different SNRs (SNR = 1, 5, 10, 20, 50) and RERs (RER = 1.36, 1.64, 1.82, 2). We can see that the sources can be resolved with all the four RERs in the case of SNR = 50, however, the distortion becomes larger with the increase of RER. With SNR varying from 5 to 10, the two-point sources with RERs of 1.36, 1.63, and 1.82 can be resolved, while that with RER = 2 cannot be resolved. For even smaller SNR, the two-point sources cannot be resolved anymore with all the four given RERs. It means that the noise level of the images for reconstruction influences the resolving power enhancement of AMIE seriously. Fortunately, we can still obtain ~1.8 times resolving power enhancement with SNR of ~10. To study the distortion of AMIE images induced by noise, mean squared error (MSE) is calculated between the normalized IDL and normalized AMIE images. To avoid the influence of the artifacts in background (see the blue rings in Fig. 3(a)), the MSE is calculated from a 17 × 27 pixels window at the center of images (see the small dashed white window in Fig. 3(a)), which just encircles the sources. The results are shown in Fig. 3(b). Indeed, the absolute value of MSE will be influenced by the field of view (or the size of the area without light) of the image, as a result, the comparison of the MSEs for different RERs may not be meaningful. However, a comparison of MSEs for the images region with different SNR will show some useful information. The results show that the MSEs decrease rapidly with the increase of SNR in the case of SNR < 10, and then become relatively stable with small variations with SNR in the range of 10 to 100. From Fig. 3, we can find that although the resolving power enhancement has decreased from ~5 times (in noise-free case) to ~1.8 times, AMIE still performs well even with SNR of only 10.

### Experimental results

**Figure 4:**
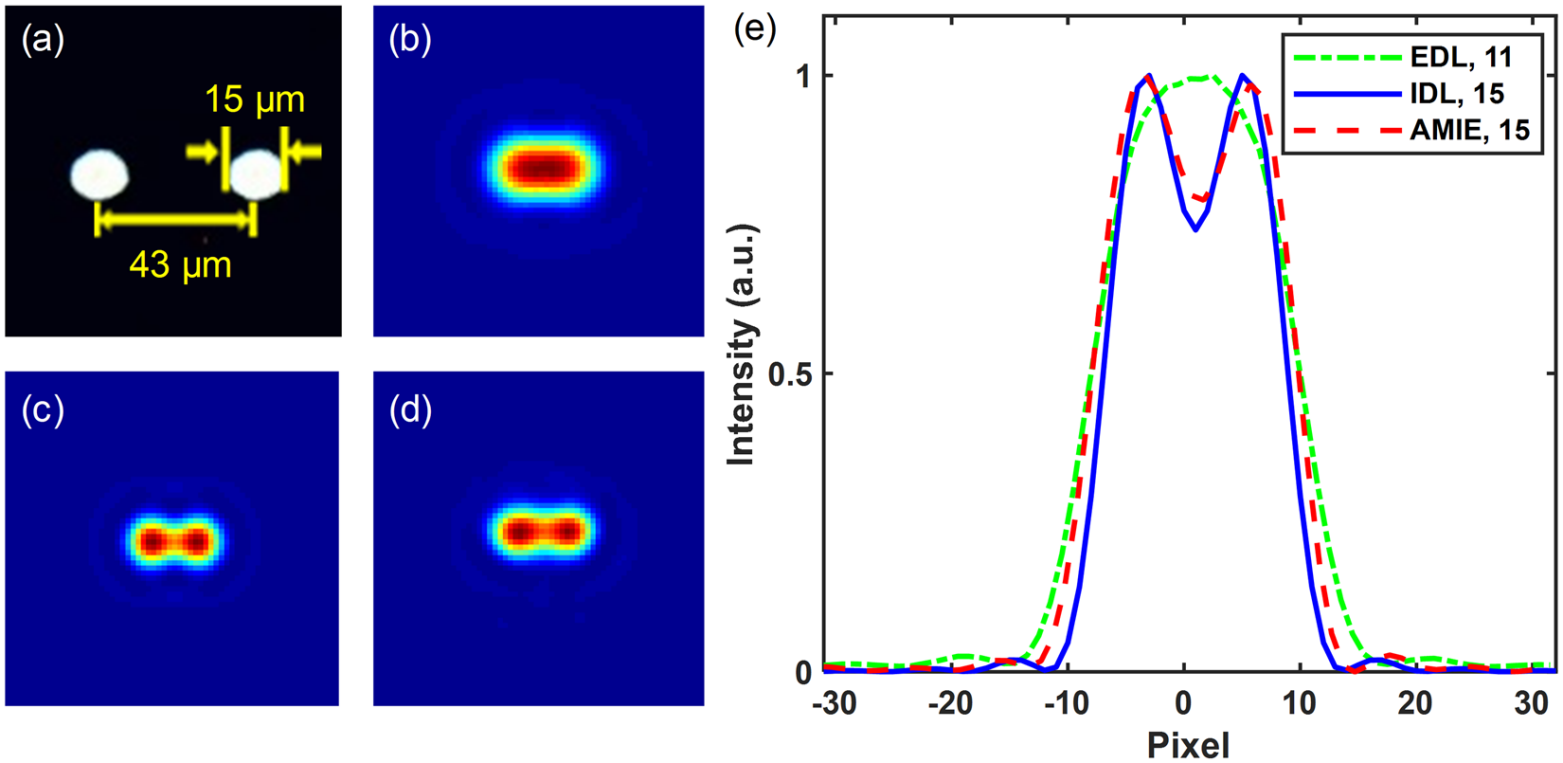
Experimental results. (**a**) The microscopic image of the two-hole target used in the experiment. (**b**) The EDL image captured with *D* = 11 mm. (**c**) The IDL image with *D* = 15 mm. (**d**) The AMIE image with *D*_AMIE_ = 15 mm. (**e**) The cross section comparison of (**b**–**d**). (EDL: experimentally captured image using diffraction-limited imaging method).

An experimental study of AMIE is performed to verify the numerical simulation results. The experimental setup is shown in Fig. 1(b). The target has two transparent holes with the diameter of 15 µm and the center-to-center distance of 43 µm, which can be just resolved with the aperture size *D* = 15 mm according to the Rayleigh criterion. The microscopic image of the target is shown in Fig. 4(a). The tunable aperture size is changed from *D* = 5.5 to 11 mm with a step size of 0.5 mm using a variable iris (the minimum scale of the variable iris is 0.5 mm in our experiment), and 12 images are captured (one image for each aperture size; the sCMOS is not saturation). With the 12 images, the intensity curve fitting and extrapolation are performed.

Figure 4(b) shows the experimentally captured image using the experimental diffraction-limited (EDL) imaging system with *D* = 11 mm and the SNR of the image is ~200. Figure 4(c,d) are the IDL and AMIE images with *D* = 15 mm and *D*_AMIE_ = 15 mm, respectively. The cross section comparison of Fig. 4(b–d) is shown in Fig. 4(e). The results indicate that the target, which cannot be resolved by EDL with *D* = 11 mm (green dash-dot line), is clearly resolved by AMIE (red dashed line). However, slight distortion and ~13% broadening of the intensity curve are observed. The distortion is mainly due to the displacement between the captured image sequence, and a simulation is given in the Supplementary Information, with the main results shown in Figs S8,S9. In the experiment, we adjust the aperture and accumulate the images manually, as a result, it takes ~5 minutes to finish the data accumulation. In this case, the vibration of the table and the airflow may cause unavoidable fluctuation of the imaging system and then shift the position of images. The shifts, or offsets, of the images are mainly in sub-pixel level on the imaging sensor, which are difficult to be rearranged perfectly. Unfortunately, this sub-pixel offsets of the images influence the performance badly, especially when the extrapolated aperture *D*_AMIE_ is larger than 16 mm, as shown in Fig. S8. Since the image offsets larger than one pixel can be registered conveniently with some well-developed methods^22^, using an imaging sensor with smaller pixel size will help to enhance the performance of AMIE, as shown in Fig. S9. More details of the simulated results are given in the Supplementary Information.

## Discussion

The cut-off frequency is determined by the aperture size and higher frequency components beyond *f*_cutoff_ will not be collected directly by common telescopic imaging system. The SR method demonstrated in this paper, i.e. AMIE, can recover frequency components beyond *f*_cutoff_ of the imaging system, and then reconstruct the SR image. It should be noticed that AMIE aims to break the optical diffraction limit of the imaging system, and it is not suitable for the improvement of the resolution-limit induced by insufficient sampling of the imaging sensor.

The key point of AMIE is a suitable function for the fitting of the intensity function *I*(*D*) of the aperture on the image plane. We have tried different functions, e.g. polynomial function, however, the performance (resolving power enhancement, distortion, etc.) is not good enough because these function are not accurate approximations of the real function *I*(*D*). The function used in this paper is a summation of a series of functions {*I*_*i*_(*D*)}, which makes the fitting, extrapolation and reconstruction more flexible. We can increase the number of functions {*I*_*i*_(*D*)} (also the number of captured images for reconstruction) in the case of complex objects, and the number of the functions can be reduced largely in the case of simple objects. However, we think there should be a more suitable fitting function than the one used in this paper, and even higher performance of AMIE may be obtained.

With the fitting function demonstrated in this paper, AMIE can enhance the resolving power of a diffraction-limited imaging system by >5 times in the ideal situation. However, the noises and the offsets of the images will reduce the performance of AMIE. Actually, other SR reconstruction algorithms also suffer from the same problem of image offsets when image sequence is involved. Various algorithms have been proposed to register the images to sub-pixel level offsets^22^. The simulation results in Supplementary Information reveal that imaging sensor with smaller pixel size or images with higher sampling rate will help to reduce the negative impact of offsets on the performance of AMIE. The noise of the images is assumed to be white Gaussian in this paper, and a low-pass filter is used for image pre-processing. However, in practical astronomical observation, the captured images are usually contaminated by different kinds of noises. Since there is no single algorithm that can deal with all noise scenarios, more suitable filters should be adopted or designed to reduce noise^23^. The noise of all the images used for SR reconstruction may have the same probability distribution function, but that of each pixel in one image is somewhat random. It means that the errors of the fitted and extrapolated intensity function induced by the noise vary with image pixels. This kind of errors will introduce distortions to the reconstructed AMIE image, for example, the broadening, shrinking and other kind of deformation of characteristic structures. The asymmetric distribution of the AMIE images in Fig. 3(a) is probably caused by this kind of errors. Considering that the noise is a stochastic variable, the problem may be solved by introducing statistical methods to AMIE.

It should be noticed that AMIE seems only suitable for space-borne telescopes or ground-based telescopes with high-performance adaptive optics system. The dynamic aberrations induced by atmospheric turbulence will impose dynamic distortions to the image sequence, and make the fitting and extrapolation of the intensity function a tough task. In practical applications, the AMIE system can be encapsulated into a compact device and connected to the exit pupil of these telescopes by relayed optics. An electronic aperture modulator will help to improve the imaging speed and the accuracy of the aperture size adjustment.

In conclusion, a novel SR imaging method with compact and low-cost setup is demonstrated. It can break the optical diffraction limit of a telescopic imaging system and improve the resolving power by >5 times in the noise-free case. AMIE opens a new horizon for SR telescopic imaging.

## Methods

### Fabrication of targets

The two-hole target, which has transparent holes with diameter of 15 µm and center-to-center distance of 43 µm, is obtained by direct laser machining of a thin aluminum film coated on a fused silica glass plate. The microscopic image of the target is shown in Fig. 4(a).

### Typical procedure of AMIE

**Figure 5:**
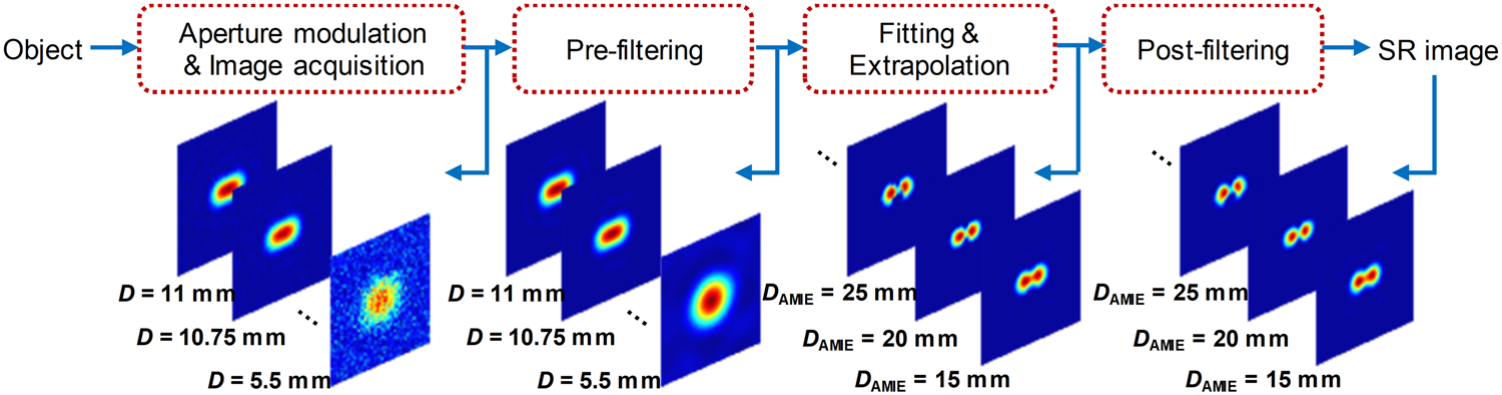
Typical procedure of AMIE.

Figure 5 demonstrates the typical procedure of AMIE which includes four steps: (1) Aperture modulation and image acquisition: a sequence of images are obtained by the same imaging system but different aperture sizes. (2) Pre-filtering: the sequence of captured images are pre-filtered according to *f*_cutoff_ of the corresponding aperture sizes. (3) Intensity curve fitting and extrapolation: a fitting of the intensity function on a position/pixel of the image plane is performed with the pre-filtered image sequence, and then the fitted curve is extrapolated to obtain the intensity value beyond the maximum aperture. The processes of fitting and extrapolation are performed for all the positions/pixels on the image plane, consequently the extrapolated image is reconstructed. (4) Post-filtering: a post-filtering is performed to filter out the frequency components beyond the *f*_cutoff_ of the extrapolated aperture size, and then an SR image is obtained.

### Pre- and post-filtering

Noise in the images will influence the fitting, extrapolation and then the SR image reconstruction. Therefore, a pre-filtering process is necessary to reduce the influence of the noise. Similarly, the residual noise will also induce artifacts and distortions to the reconstructed SR image, and a post-filtering may help to improve the image quality. The design of the filter is based on the fact that the frequency components beyond *f*_cutoff_ of the aperture size mainly come from the noise. The pre- and post-filter is described as.

10 $${H}({f})=\{\begin{array}{l}0,\,f > {{f}}_{{\rm{cutoff}}}\,{\rm{of}}\,{\rm{aperture}}\,{\rm{size}}\,{\rm{or}}\,{\rm{extrapolated}}\,{\rm{aperture}}\,{\rm{size}}\\ 1,\,\text{otherwise}\end{array}$$

### Curve fitting

**Figure 6:**
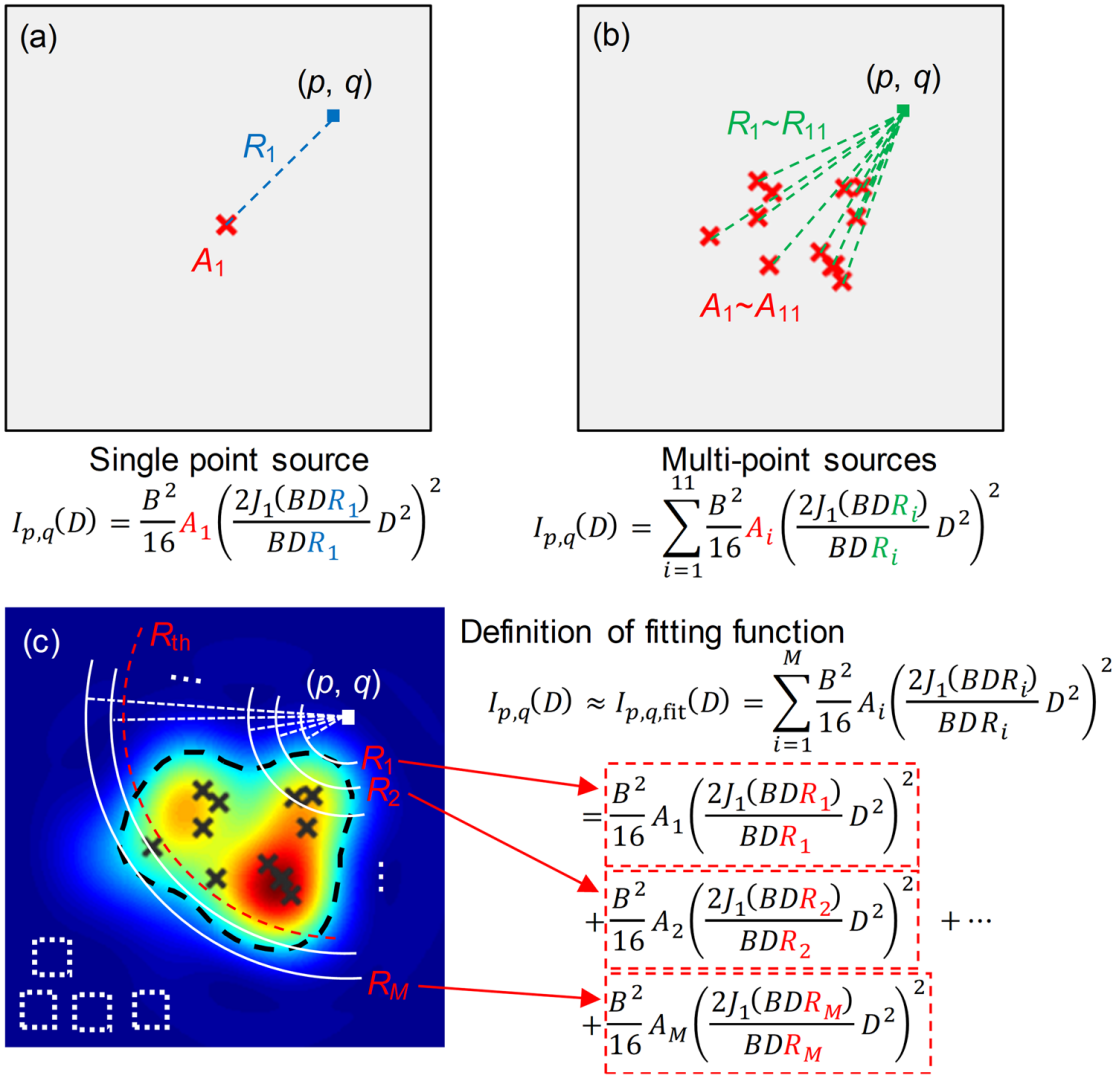
Illustration of the definition of {*R*_*i*_} and fitting function. (**a**,**b**) The case of single point source and multi-point sources, respectively. The red crosses show the positon of the geometric image of the sources. (**c**) The definition of {*R*_*i*_} and fitting function of pixel (*p*, *q*). The dashed black curve shows the contour of the image of the multi-point sources in (**b**). The circular arcs are concentric, and the center is pixel (*p*, *q*). The radii of these arcs have a uniform interval. The white dashed windows are randomly placed far away from the contour, which are used for sampling the noise intensity.

Figure 6(a,b) show the *I*_*p*,*q*_(*D*) in the case of single point source and multi-point sources (without noise), respectively. As we can see, the *I*_*p*,*q*_(*D*) is a summation of many terms, and each term corresponds to a different geometric image of point source with its own *A*_*i*_ and *R*_*i*_. Therefore, In order to obtain the most accurate approximation of the true intensity function of a given pixel (*p*, *q*), the fitting function *I*_*p*,*q*,fit_(*D*) should have the same form as the *I*_*p*,*q*_(*D*), i.e. a summation of many terms with their own {*A*_*i*_} and {*R*_*i*_}. Since the structure information of the object is unknown, the method by assuming each pixel on the image plane as a term is hard to operate in practice. To handle this problem, here we define the term as a combination of all the point sources that have the same distance *R*_*i*_ away from the position (*p*, *q*), as shown in Fig. 6(c). The fitting process is to estimate {*A*_*i*_} and {*R*_*i*_} with a series of measured data {*I*_*p*,*q*_(*D*)}. However, it is still a difficult task to estimate {*A*_*i*_} and {*R*_*i*_} simultaneously, because the search space is too large and few constrains are applied. The solution is proposed as follow: firstly, we manually define {*R*_*i*_} according the information extracted from the acquired images; then, {*A*_*i*_} is estimated with fixed parameters {*R*_*i*_} by constrained least-squares minimization. Actually, from a theoretical point of view, the more the terms are, the more accurate the approximation is. However, the number of acquired images is limited by the precision of the aperture modulator or the imaging speed. As a result, the number of the terms is usually set as the number of acquired images in this paper.

Figure 6(c) shows the process of defining {*R*_*i*_}. Firstly, the pre-filtered image with the maximum aperture size *D*_max_ is segmented into two part: the part with light irradiation (we call it foreground for short), and the part where light irradiation can be neglected (we call it background for short). The geometric image of the object is assumed underneath the foreground. The segmentation can be performed manually or by algorithm^24^. Then, the {*R*_*i*_} can be defined according to the distances between pixel (*p*, *q*) and the foreground. If *M* images with different aperture sizes are acquired, then the {*R*_*i*_} can be defined by a uniform interval.

11 $${{R}}_{{i}}={{R}}_{1}+({i}-1)\times \frac{{{R}}_{{M}}-{{R}}_{1}}{{M}-1},\,({i}=1,2,\,\cdots \,,{M}),$$

where *R*_1_ represents the minimum distance and *R*_*M*_ represents the maximum one, as shown in Fig. 6(c) marked with white circular arcs, and all the geometric image of the multi-point sources marked by black crosses is inside the foreground. If pixel (*p*, *q*) is inside the foreground, then *R*_1_ is zero.

In the case of noisy images used for reconstruction, the treatment of {*R*_*i*_} becomes a little more complicated. As shown in Fig. 6(c), for example, if a given pixel (*p*, *q*) is too far away from the foreground, then for *R*_*i*_ that is larger than a threshold *R*_th_ (marked with red dashed circular arc), the true signal intensity of each term may be drown in the noise. Consequently, the estimated parameters will become obviously unreliable and cause unpredictable distortions. Thus, these corresponding terms should be removed by modifying *R*_*M*_ to *R*_th_. The *R*_th_ is computed as follows.

12 $${{I}}_{{\rm{fgmax}}}{(\frac{2{{J}}_{1}({B}{{D}}_{{\rm{\max }}}{{R}}_{{\rm{th0}}})}{{B}{{D}}_{{\rm{\max }}}{{R}}_{{\rm{th0}}}})}^{2}={{I}}_{{\rm{noise}}},$$

13 $${{R}}_{{\rm{th}}}={C}{{R}}_{{\rm{th0}}}$$

where *I*_fgmax_ is the maximum intensity in the foreground, *I*_noise_ is the maximum intensity in the sampling windows which is randomly placed far away from the foreground (see white dashed windows in Fig. 6(c)). Equation (12) is calculated as that the maximum intensity of the true signal intensity is equal to that of noise with *R*_th0_. However, the *R*_th0_ is found crude in the experiments. A coefficient *C* is multiplied to *R*_th0_ to achieve a further adjustment *R*_th_ as described by Eq. (13). The *C* ranges from 0 to 1, and AMIE performs well with *C* ranging from 0.65 to 0.85 in this paper empirically.

With the defined fitting function and the *M* acquired images, the {*A*_*i*_} in *I*_*p*,*q*,fit_(*D*) can be obtained by constrained least-squares minimization

14 $${\boldsymbol{A}}={\rm{\arg }}\,{{\rm{\min }}}_{{\boldsymbol{A}}}{\sum }_{{\boldsymbol{j}}=1}^{{\boldsymbol{M}}}{({{I}}_{{j}}-{\hat{{I}}}_{{j}})}^{2}{\rm{s}}{\rm{.t}}{\rm{.}}\,{\boldsymbol{A}}\ge 0,$$

where ***A*** = (*A*_1_, *A*_2_, …, *A*_*M*_)^T^, *I*_*j*_ and *Î*_*j*_ are the measured and fitted intensities of pixel (*p*, *q*) in *j*-th image, respectively.

## Electronic supplementary material

Supplementary Information

## Data Availability

The datasets generated and analyzed during the current study are available from the corresponding author on reasonable request.
