# Supplementary material for: Super-resolution imaging via aperture modulation and intensity extrapolation
Source: Sci Rep. 2018 Oct 12;8:15216. doi: 10.1038/s41598-018-33416-9 (PMC6185970; doi:10.1038/s41598-018-33416-9)
Supplement: Supplementary file 1 — Supplementary Information [file 41598_2018_33416_MOESM1_ESM.pdf]

Supplementary Information

Super-resolution imaging via aperture modulation and intensity extrapolation

Biao Xu[1,2,3], Zhiqiang Wang[1,2], and Jinping He[1,2,*]

[1]National Astronomical Observatories/Nanjing Institute of Astronomical Optics & Technology, Chinese Academy of Sciences, Nanjing 210042, China
[2]Key Laboratory of Astronomical Optics & Technology, Nanjing Institute of Astronomical Optics & Technology, Chinese Academy of Sciences, Nanjing 210042, China
[3]University of Chinese Academy of Sciences, Beijing 100049, China
[*]Corresponding author: jphe@niaot.ac.cn

1. Limit of resolving power enhancement by AMIE in the noise-free case

According to the Rayleigh criterion, the minimum spatial resolvable distance for incoherent imaging is $\Delta d = 1.22\lambda F/D$. In order to investigate the limit of resolution enhancement, simulation experiments of two-point sources with decreasing center-to-center distance (ΔL) are performed. The image sequence for curve fitting are generated with D = 5.5 to 11 mm, with a step size of 0.25 mm, the two-point source are of equal brightness, λ = 530 nm, F = 1000 mm (the same below). Figures S1 and S2 show ideal diffraction-limited (IDL) images and AMIE images with different aperture size. Figure S3 shows the cross section comparison of the images in Figs. S1 and S2 and gives the ratios of the full-width at half-maximum of AMIE image to that of IDL image (FWHM ratio). The graphs show that the extrapolated intensity distribution, compared with the IDL ones, has a slowly increasing distortion as ΔL decreases, especially, when ΔL < 11.8 µm, i.e. the D_{AMIE} > 55 mm, the FWHM ratio will exceed 1.1 and the saddle-to-peak ratio (SPR) will be slightly larger than 0.735. We consider this critical point as the limit of resolution enhancement and the resolution enhancement ratio, which is defined by the ratio of spatial resolution (RER = $\Delta d_{\text{telescope}} / \Delta d_{\text{AMIE}} = D_{\text{AMIE}} / D_{\text{telescope}}$), is ~5 times. Actually, though the extrapolated intensity distributions are distorted for D_{AMIE} > 55 mm in some degree, the two-point source can still be distinguish clearly. Therefore in the practical applications of binary-star observation, a farther aperture size may be used for extrapolation to achieve >5 times RER to resolve a binary-star system when the distortion of the disks is ignorable. Other two-point sources with similar parameters obtain the same results.

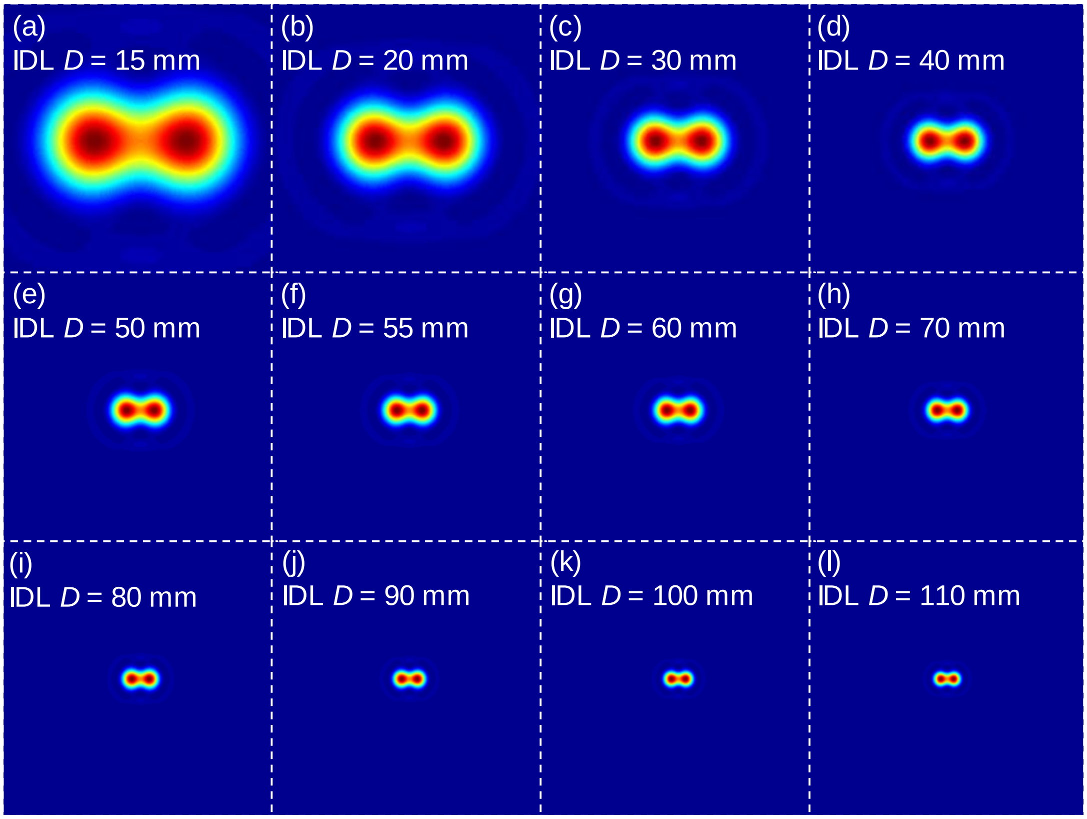

Supplementary Figure S1. IDL images with different resolvable aperture size of Rayleigh criterion.

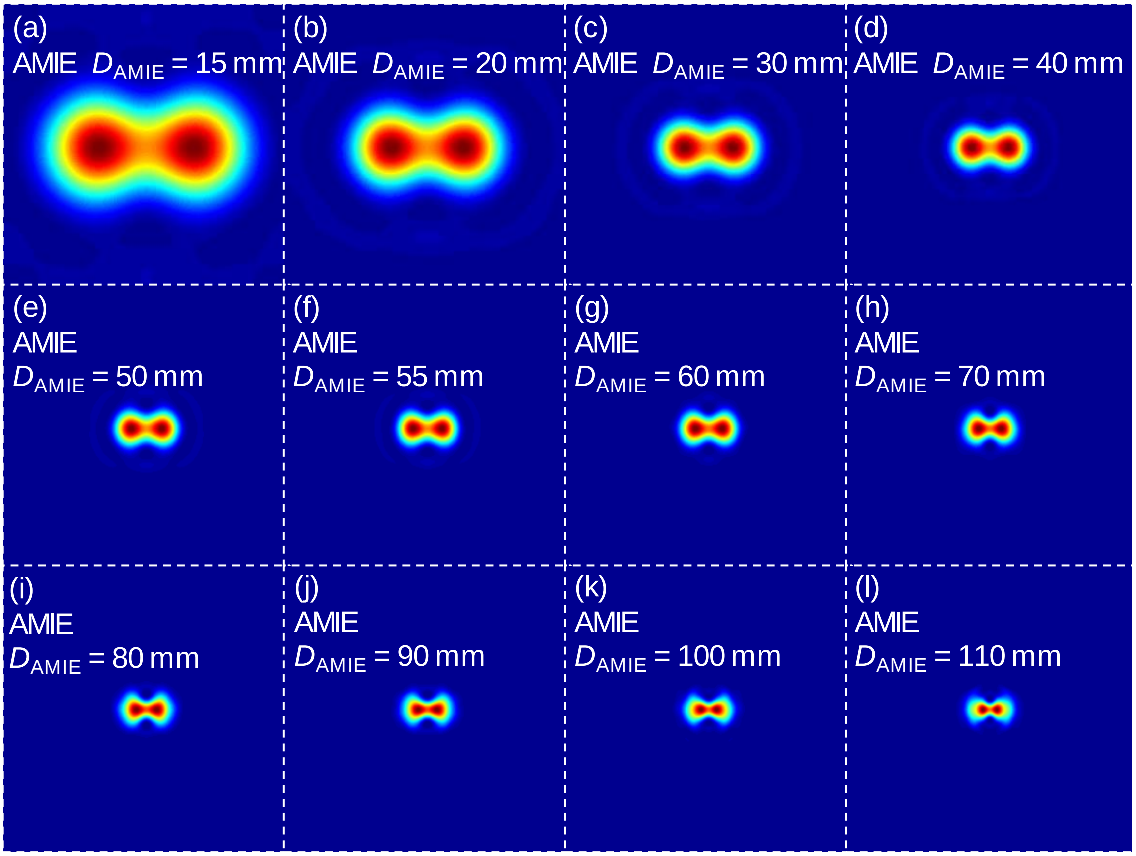

Supplementary Figure S2. AMIE images with different resolvable aperture size of Rayleigh criterion.

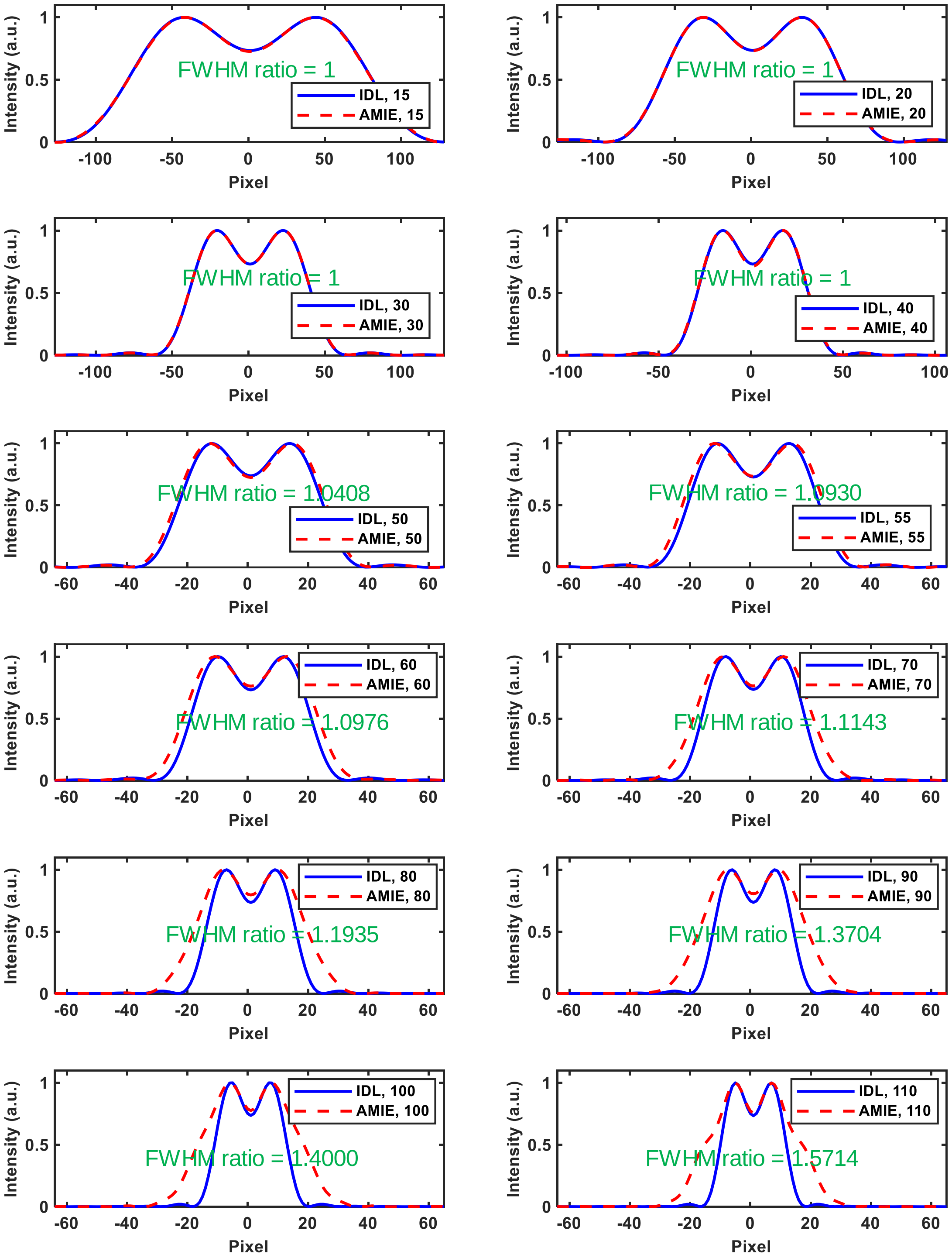

Supplementary Figure S3. Cross section comparison of the images in Figs. S1 and S2.

2. Limit of extrapolation

Due to the extrapolation distortion mentioned in part 1, it is necessary to investigate the limit of extrapolation for a specified object. In this part, a two-point source with ΔL = 43.1 μm (Rayleigh criterion of D = 15 mm) is taken as an example and no noise is added in the simulation experiments. Figure S4 shows IDL images with different aperture sizes. Figure S5 shows the AMIE images with different extrapolated aperture sizes. Figure S6 shows the cross section comparison of the images in Figs. S4 and S5. The results indicate that when D_{AMIE} > 60 mm, slight distortion will be introduced into the AMIE images. When D_{AMIE} > 150 mm, the disks will be badly distorted. Here we define the ratio of the critical D_{AMIE} to the diameter satisfying the Rayleigh criterion as the limit of extrapolation, which is ~4 times in our simulation experiments in noise-free case. Other two-point sources with similar parameters obtain the same results. Noise will weaken the limit of extrapolation badly, which is shown in Fig. S10.

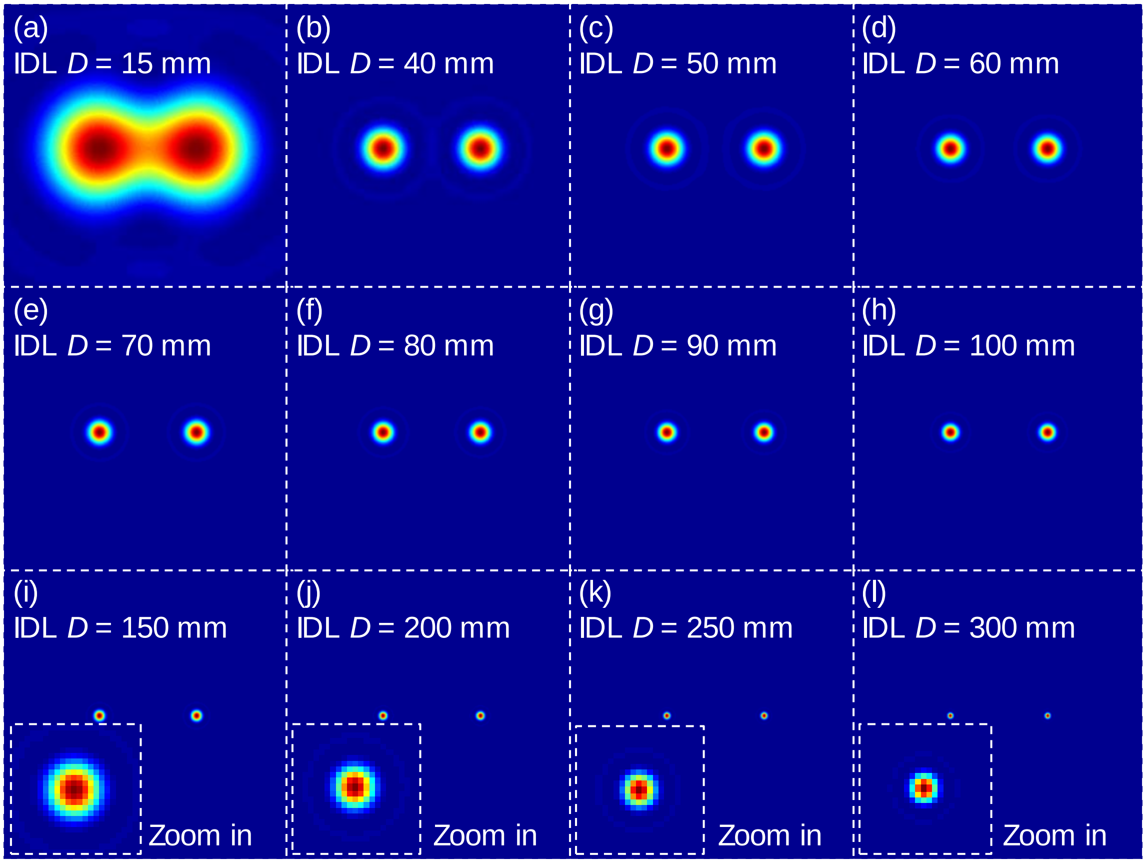

Supplementary Figure S4. The IDL images of two-point source (ΔL = 43.1 μm) with different aperture size.

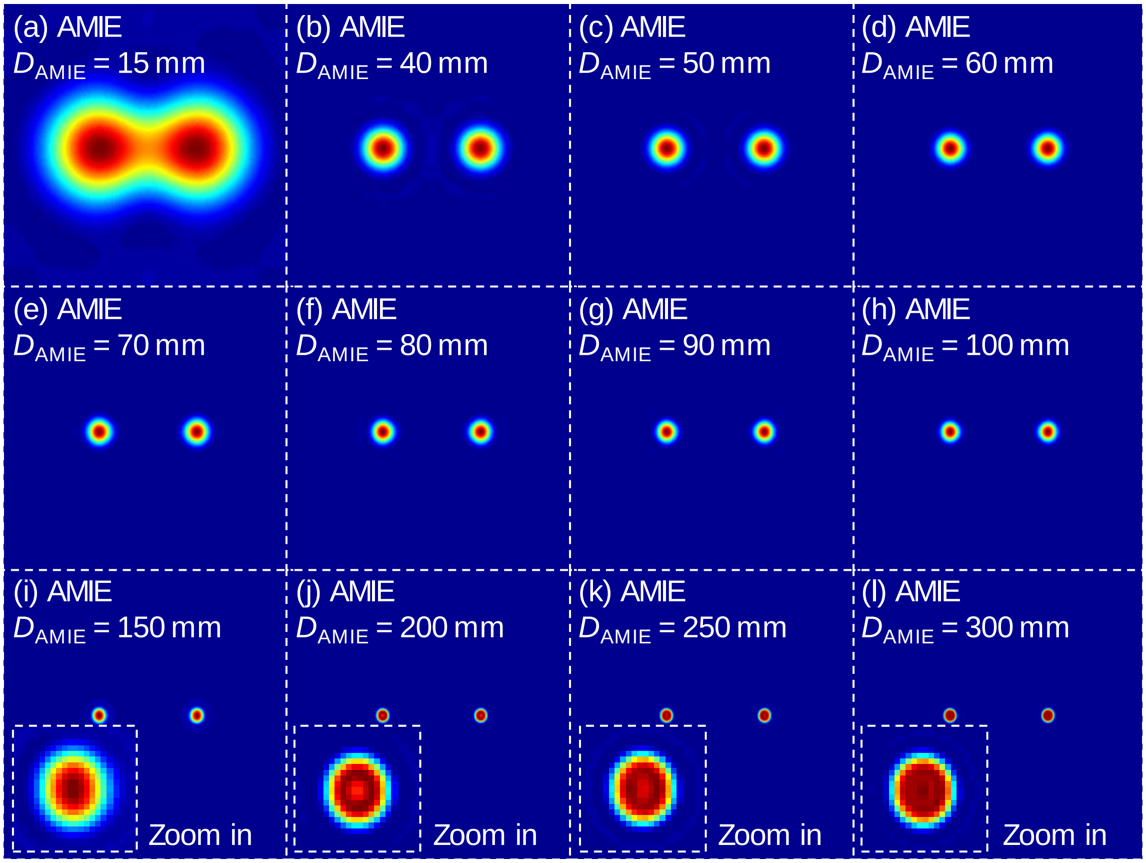

Supplementary Figure S5. AMIE images of two-point source (ΔL = 43.1 μm) with different extrapolated aperture size.

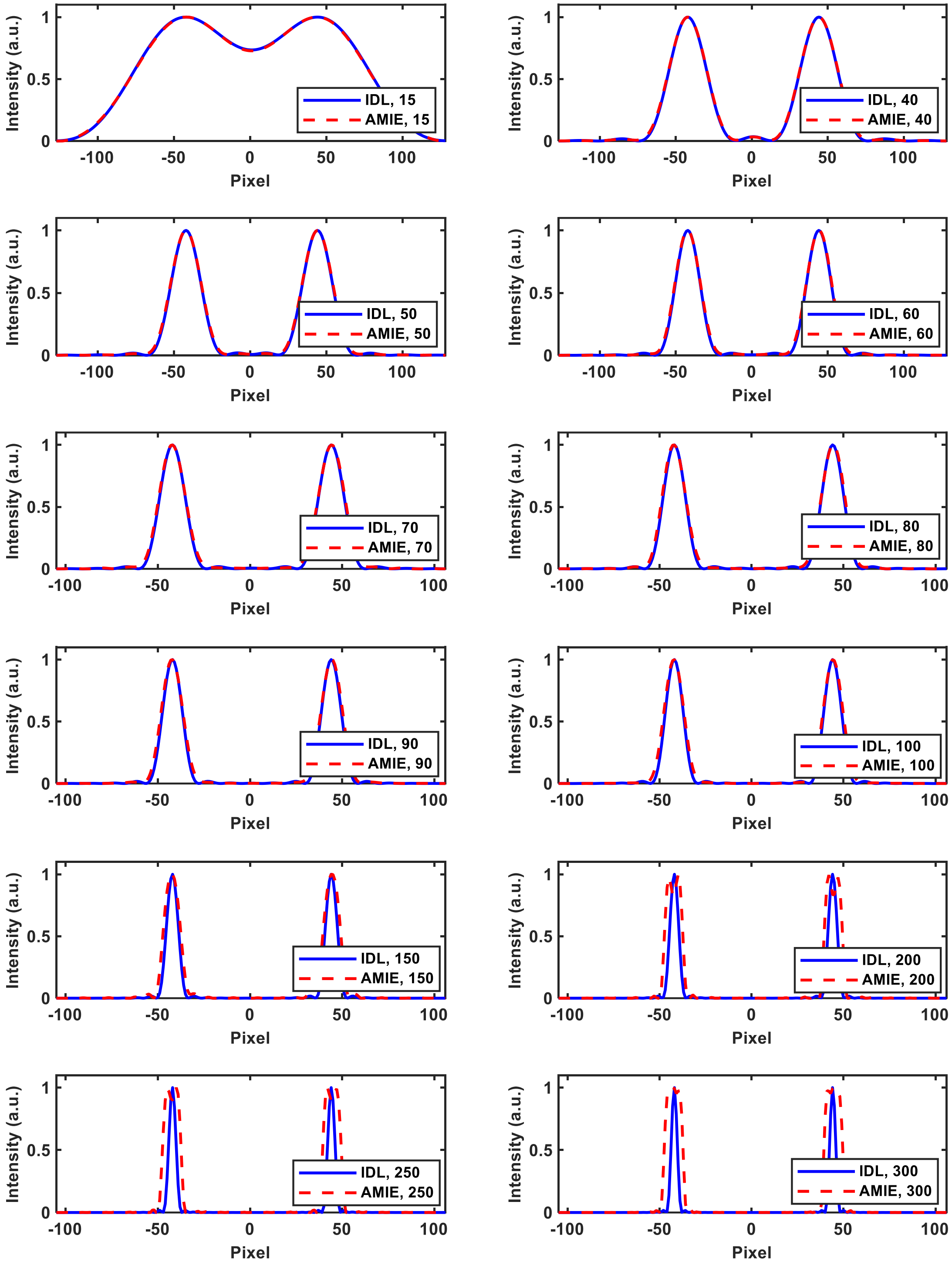

Supplementary Figure S6. Cross section comparison of the images in Figs. S4 and S5.

3. Performance of AMIE of two point sources with non-equal brightness

In this part, a two-point source with ΔL = 11.8 μm (Rayleigh criterion of D = 55 mm, RER = 5) and non-equal brightness is taken as an example to show the performance of AMIE. The parameters of simulation are the same as used above except the relative brightness of these two point sources, which are set to 1: 0.8, 1: 0.6, 1: 0.4, 1: 0.2 and 1: 0.1, respectively. We adopt the modified Sparrow criteria which is proposed in Ref. [Asakura, T. Resolution of two unequally bright points with partially coherent light. *Nouvelle Revue d'Optique* **5(3)**, 169 (1974)] for the evaluation of resolution in the case of non-equal brightness. The modified Sparrow criteria is stated as: the resolution is retained when the second derivative of the image intensity distribution vanishes at a certain point X_0 between two peaks, with the condition that this point X_0 should be a solution for the first derivative of the image intensity distribution becoming zero. Simple version of the modified Sparrow criteria: the two point sources are considered resolved when the intensity between the peaks shows a minimum. The blue curves in Fig. S7 show that the two-point source with different ratio of brightness can be resolved according to the modified Sparrow criteria. The red dashed curves are the AMIE results and they agree well with the blue ones, in spite of slight broadening. Similarly, as demonstrated foregoing, the presence of noise will weaken the performance of AMIE, especially when the difference between the peaks becomes great in the case of small SNR, because the low peak will be covered by noise. The results show that AMIE performs well in the case of two-point source with non-equal brightness.

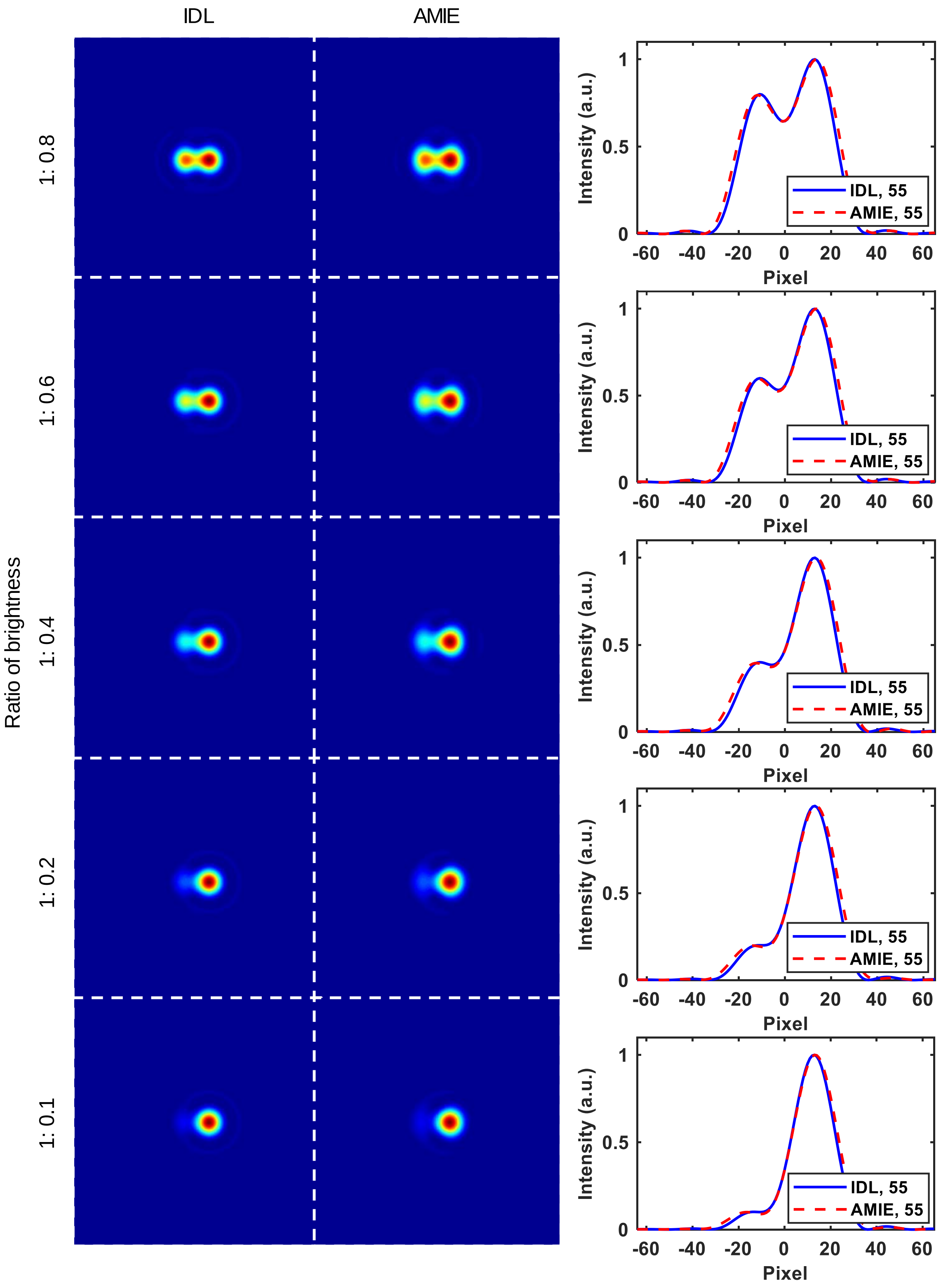

Supplementary Figure S7. The IDL and AMIE images of two-point source (ΔL = 11.8 μm) with different ratio of brightness, no noise added.

4. Effect of the offsets on the performance of AMIE

In the experiments, we find that the platform vibration and airflow will cause small but random offsets corresponding to different image capturing process, which badly influence the fitting and extrapolation results of AMIE. Moreover, the offsets are often sub-pixel level as well as multi-pixel level in practical applications. With the help of image registration algorithms, the multi-pixel offsets can be corrected so that only sub-pixel offsets are considered in the simulation experiment. We still take a two-point source with ΔL = 43.1 µm as an example to investigate the impact of offsets on the performance of AMIE and noise is not considered in this section. Figures S8 and S9 show the AMIE images with pixel size of 5.04 µm and 1 µm when random sub-pixel offsets (< half of pixel size) are added into the image sequences, respectively.

The results show that the target cannot be resolved even if the image is extrapolated to D_{AMIE} = 20 mm when the pixel size is 5.04 µm at the image plane, but the same target, captured with smaller (1 µm) pixel size, is clearly resolved at D_{AMIE} = 15 mm with tiny distortions caused by the offsets. For other two-point sources with smaller ΔL, a much smaller pixel size is required. The simulation results indicate that, after the multi-pixel level offsets are corrected by the image registration algorithms, the impact of sub-pixel offsets can be reduced by using a camera with smaller pixel size (increasing the sampling rate of sensor). Consequently, the higher the sampling rate is, the better the performance of AMIE will be.

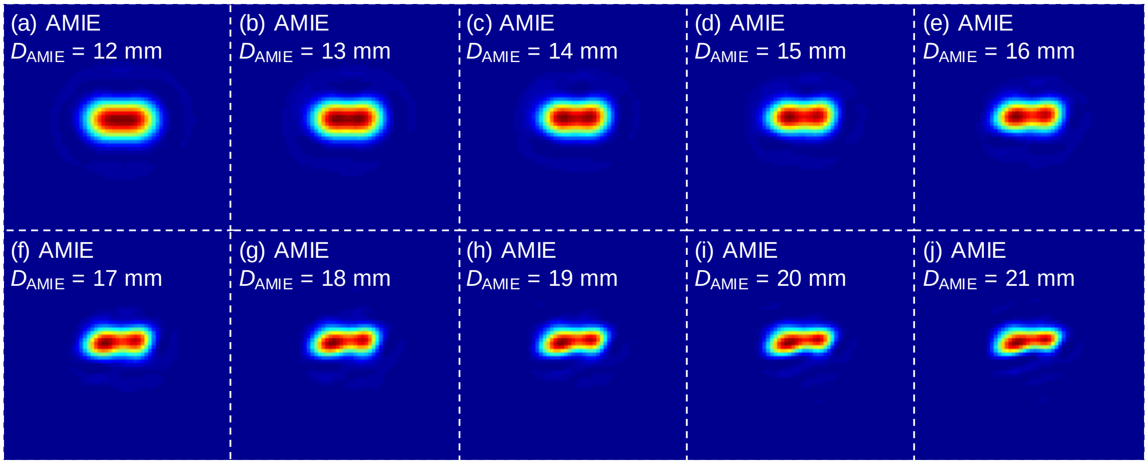

Supplementary Figure S8. The AMIE images of two-point source (ΔL = 43.1 µm) with different extrapolated aperture size (Pixel size = 5.04 µm).

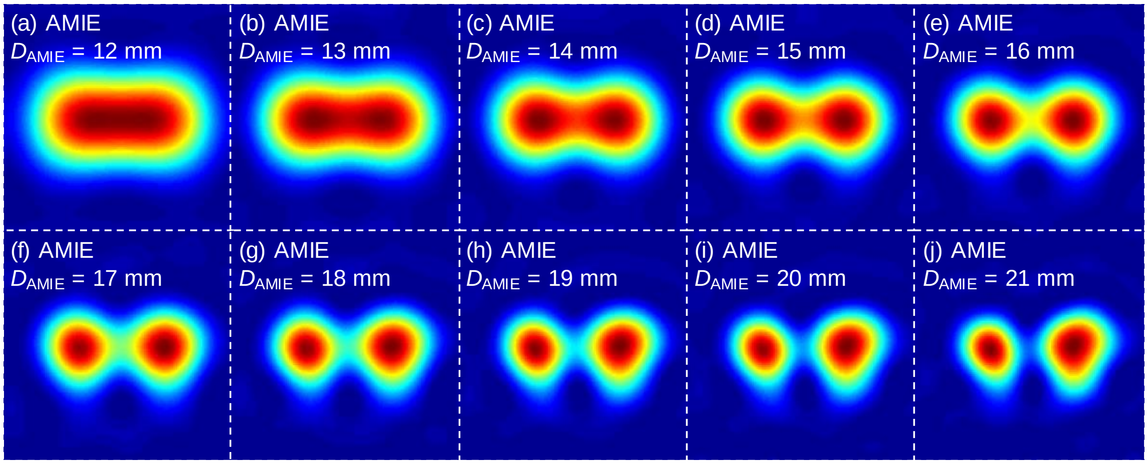

Supplementary Figure S9. The AMIE images of two-point source (ΔL = 43.1 μm) with different extrapolated aperture size (Pixel size = 1 μm).

5. Discussion of the coefficient *C*

A simulation study of two-point source with ΔL = 43.1 μm is performed to investigate the effect of *C* on the AMIE result when white Gaussian noise is added with SNR = 10. Figure S10 displays some of the AMIE images with different extrapolated aperture sizes and coefficient *C*. It confirms that noise will significantly influence extrapolation results of AMIE. To different extrapolated aperture size D_{AMIE}, the coefficient *C* leading to the best extrapolation performance varies at a quite large interval (AMIE performs well with *C* ranging from 0.65 to 0.85 in Fig. S10), a compromise should be made between the resolution enhancement and distortion in fitting and extrapolation process. Adaptive *C* may help to improve the performance of AMIE, which is still under study.

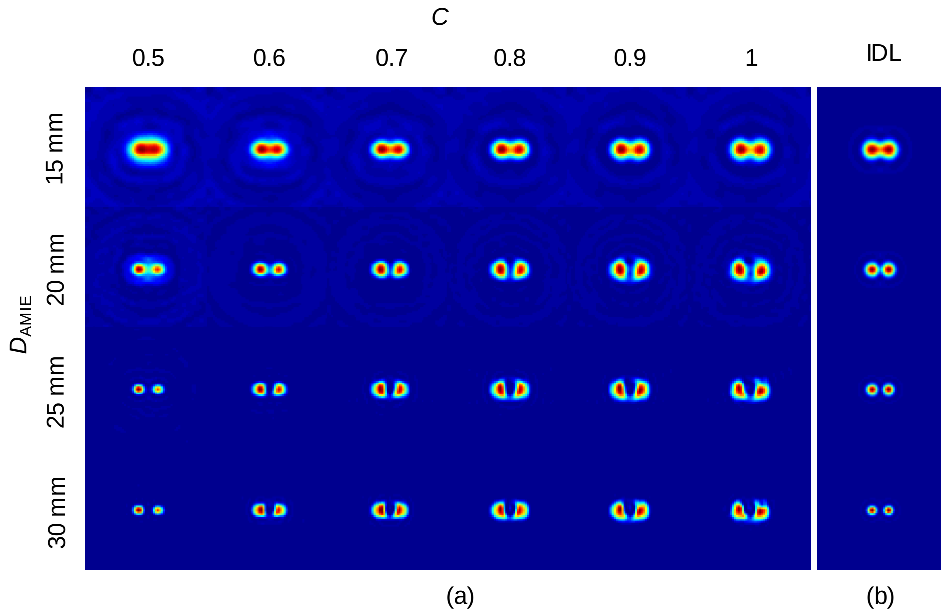

Supplementary Figure S10. Some of the AMIE images with different extrapolated aperture sizes and coefficient *C*.
